# Hope for 17 patients with chronic cluster headache: efficacy evaluation of upper cervical spinal nerve root release surgery (2020–2023)

**DOI:** 10.3389/fneur.2025.1662677

**Published:** 2025-11-10

**Authors:** Yang Mao-jiang, Qiong Xian, Yang Hong-ying, Zhang Han-wen, Qiu Zhi-qiang, He Li-bing, Mohd Uzid, Xu Xiao-xue, Husni Ahmed Abdullah Al-Goshae

**Affiliations:** 1 Department of Pain, Affiliated Hospital of North Sichuan Medical College, Nanchong, Sichuan, China; 2 School of Graduate Studies, Post Graduate Centre, Management and Science University (MSU), Shah Alam, Selangor, Malaysia

**Keywords:** cluster headache, spinal cord, upper cervical spine, neurolysis, CT

## Abstract

**Objective:**

We aimed to evaluate the effectiveness and safety of upper cervical spinal nerve root release surgery in the treatment of chronic cluster headache.

**Method:**

This retrospective study reviewed 17 patients diagnosed with chronic cluster headache who underwent upper cervical spinal nerve root release surgery between August 2020 and March 2023. Data collected included demographic information, headache characteristics (frequency, duration, and intensity), preoperative treatment regimens, and postoperative outcomes. The surgical procedure aimed to alleviate nerve compression in the upper cervical region to improve neurological function. Patients were routinely followed postoperatively to assess the therapeutic impact through headache symptom relief assessments, including headache impact tests, pain scores, and quality of life evaluations.

**Result:**

All 17 patients completed the surgery without severe complications. Our 6-month follow-up data demonstrated notable improvements in headache symptoms, with a significant reduction in the frequency and duration of headache episodes and alleviation of pain intensity. Specifically, the Visual Analog Scale (VAS) scores at 1 week, 1 month, 3 months, and 6 months post-surgery were (2.76 ± 1.16), (2.25 ± 0.45), (1.95 ± 0.47), and (1.75 ± 0.48), respectively. The frequency of headache episodes decreased to (1.96 ± 0.42), (1.45 ± 0.36), (0.95 ± 0.32), and (0.76 ± 0.28) times per month, respectively. The duration of each episode was reduced to (14.68 ± 4.75), (9.44 ± 3.28), (6.65 ± 2.52), and (4.55 ± 1.34) minutes, respectively. Moreover, patients reported significant enhancements in quality of life and resumed normal work and social activities.

**Conclusion:**

The findings from this case series suggest that upper cervical spinal nerve root release surgery is a safe and effective treatment for chronic cluster headache, offering substantial clinical improvements. However, due to the small sample size, further large-scale, placebo-controlled studies are essential to corroborate these results, validate the long-term efficacy and safety of the procedure, and accurately determine the contribution of the surgical intervention beyond the placebo effect.

## 1 Introduction

Cluster headache, a primary neurovascular disorder, manifests as excruciating pain localized to one eye socket, orbital, and/or temporal region. This pain is often accompanied by ipsilateral autonomic symptoms, including conjunctival congestion, tearing, nasal congestion, rhinorrhea, eyelid edema, forehead and facial sweating, miosis, and upper eyelid ptosis (1–3). Characterized by its periodicity and clustering, cluster headaches inflict significant distress on patients, severely impacting their quality of life. Current therapeutic strategies encompass medications (e.g., oxygen inhalation, triptans, and verapamil), nerve blocks, and neuromodulation. However, some patients either respond poorly to these treatments or are unable to tolerate their side effects, becoming medically refractory (4, 5). Consequently, the exploration of new effective treatment modalities remains critical for enhancing patient outcomes.

The pathophysiology of cluster headache remains elusive but is thought to involve factors such as activation of the trigeminal nervous system, hypothalamic dysfunction, and neurotransmitter imbalances (6, 7). A pivotal anatomical and functional entity in the pathogenesis of refractory headaches is the trigeminocervical complex (TCC), which comprises the caudal subnucleus of the trigeminal nucleus and the dorsal horn gray matter of the upper cervical spinal cord (C1-C2). This complex is closely linked to the trigeminal nucleus and receives afferent fibers from both the trigeminal nerve and the upper cervical spinal nerves (C1-C2), facilitating the transmission and modulation of pain signals (8–11). It is hypothesized that targeted therapy of the nerve roots in the upper cervical spine may modulate TCC activity, thus reducing trigeminal nerve excitability and alleviating pain.

In recent years, various interventions targeting the upper cervical spine have been explored. Notable among these are neuromodulation techniques such as occipital nerve stimulation (12, 13) and upper cervical spinal cord stimulation (SCS), which have demonstrated efficacy in alleviating symptoms of cluster headache (14–16). However, these treatments have limitations; for instance, nerve blocks may offer only temporary relief, while implantable devices like SCS are more invasive, costly, and carry risks of hardware-related complications. In this context, upper cervical spinal nerve root release, a minimally invasive technique, emerges as a promising alternative. By directly engaging the nerve roots of the upper cervical spinal cord, this surgical approach may offer more direct control over nerve function to achieve sustained headache relief with a potentially quicker recovery and lower risk profile compared to more invasive neuromodulation procedures.

Therefore, this study aimed to evaluate the efficacy and safety of upper cervical spinal nerve root release as a targeted surgical intervention for patients with medically refractory chronic cluster headache. We have analyzed a case series of 17 patients who underwent the procedure between August 2020 and March 2023, assessing its therapeutic impact and safety, and contributing novel clinical insights into the management of this debilitating condition.

## 2 Materials and methods

### 2.1 General information

This retrospective study collected clinical data from patients diagnosed with *chronic cluster headache* who underwent upper cervical spinal nerve root release surgery at the Pain Department of Chuanbei Medical College Affiliated Hospital, spanning from August 2020 to March 2023. Eligibility for participation was determined based on several inclusion and exclusion criteria.

#### 2.1.1 Inclusion criteria

All 17 patients strictly met the diagnostic criteria for chronic cluster headache (CCH) as defined by the International Classification of Headache Disorders, 3rd edition (ICHD-3) (2). This included experiencing attacks lasting 15–180 min with associated autonomic symptoms for a period of at least 1 year, with remission periods shorter than 3 months. Furthermore, all included patients had a history of inadequate response or intolerance to multiple pharmacological treatments (including at least three classes of prophylactic medications, such as verapamil and triptans) or to other neuromodulatory interventions like nerve blocks. Consent to undergo upper cervical spinal nerve root release surgery required participants to sign an informed consent form.

#### 2.1.2 Exclusion criteria

The following exclusion criteria applied: (1) Presence of intracranial space-occupying lesions, cerebrovascular diseases, traumatic brain injuries, or any other conditions that could induce headaches; (2) History of neck surgery or neck trauma, which might compromise surgical outcomes or elevate surgical risks; (3) Patients with primary cervical pathology, such as severe cervical spondylosis or disc herniation identified as the main source of pain, were excluded to specifically isolate the therapeutic effect on CCH; (4) Presence of severe systemic diseases such as cardiac, pulmonary, hepatic, renal dysfunctions, or mental illnesses that could impede cooperation with treatment protocols and follow-up assessments.

## 3 Methods

### 3.1 Preoperative preparation

Upon admission, each patient underwent a thorough diagnostic assessment that included computed tomography (CT) and magnetic resonance imaging (MRI) of the head and cervical spine. These imaging procedures are essential for accurately identifying any lesions within the central nervous system that could impact the surgical approach or outcome. Additionally, routine preoperative evaluations were conducted to ensure there were no contraindications to surgery. This preparatory phase is designed to optimize patient safety and surgical efficacy by addressing potential risks and complications before proceeding with surgery.

Preoperative pain assessment was carried out as follows using a custom-designed 10 cm (100 mm) visual analog scale (VAS) ruler, featuring standardized anchors at both extremities: “No pain” (0 points) at the left terminus and “Maximum imaginable pain” (10 points) at the right terminus. Assessments were conducted in a controlled environment ensuring patient comfort and communicative readiness. Prior to evaluation, patients received standardized instructions regarding VAS interpretation and marking procedures. Participants were instructed to place a perpendicular mark along the linear scale reflecting their subjective pain intensity. Scoring methodology was carried out as follows. The distance between the “No pain” anchor and the patient’s mark was measured in centimeters, with proportional conversion to a 0–10 numerical scale. For instance, a mark 3 cm from the pain-free end corresponds to a VAS score of 3. Continuous validation was maintained through endpoint confirmation—unmarked endpoints received default scores (0 or 10) while intermediate positions were calculated through metric interpolation.

### 3.2 Preoperative instructions and surgical procedure

Patients were required to fast for 6 h prior to the surgery. The entire operation was conducted by a chief physician with over 30 years of experience in interventional procedures. The Philips 64-row spiral CT served as the guiding device throughout the procedure.

#### 3.2.1 Positioning and imaging

**Figure 1:**
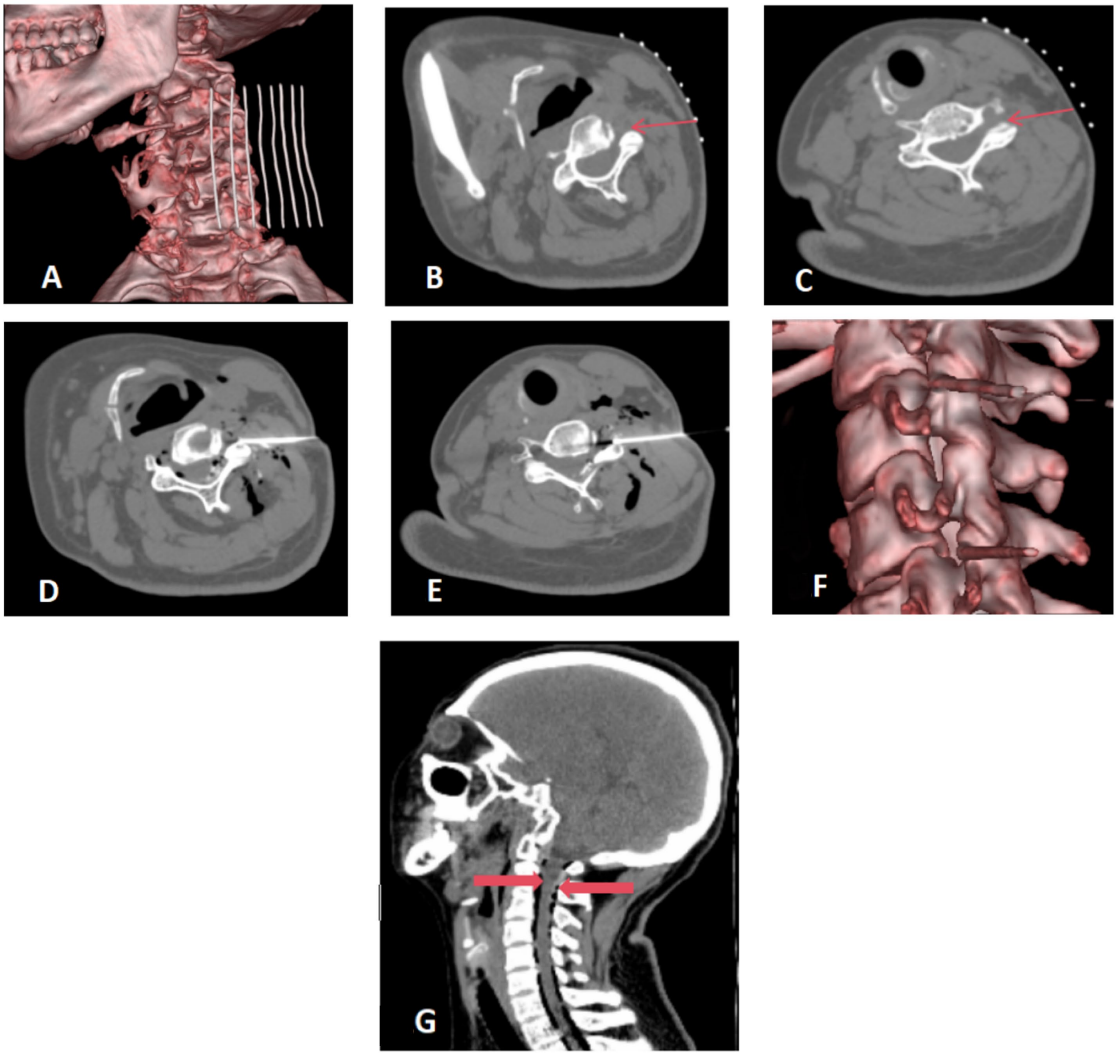
**(A–C)** Preoperative positioning: the patient was positioned supine on the operating table, with the head turned towards the contralateral side. A metal grid marker was strategically placed on the neck to facilitate precise targeting. The cervical anterolateral approach was employed, focusing on the articular pillar surfaces at the C3/4 and C5/6 levels. These target areas were distinctly marked with fine red arrows for clear visualization. **(D–F)** Needle placement and injection. The needle was precisely inserted along a pre-established pathway targeting the surface of the superior articular process. A therapeutic mixture consisting of 10 mL of ozone blended with physiological saline, complemented by 1 mL of contrast agent, was meticulously injected at each designated site. Virtual Reality (VR) reconstruction techniques were utilized to confirm the accurate positioning of the needle tip at the level of the intervertebral foramen. **(G)** Postoperative CT scan reconstruction. The sagittal view of the cervical spine after surgery exhibits a satisfactory dispersion of ozone throughout the spinal canal. Gas shadows, clearly surrounding the dura mater, are marked by thick red arrows. There are no indications of hemorrhage detected in the visualized areas.

Patients were positioned supine on the CT examination table, with their heads turned toward the unaffected side. A custom-made metal fence was attached to the upper neck area. A detailed CT scan of the neck was performed with a slice thickness of 1.0 mm, effectively mapping the area for surgical intervention (Figure 1A). Using the anterolateral cervical approach, the physician accurately located the unilateral intervertebral foramen at the C3/4 and C5/6 level. The puncture path and angle were meticulously set (Figures 1B,C).

#### 3.2.2 Surgical procedure

Following routine disinfection and draping, local anesthesia was administered using 1% lidocaine. A 14 cm long coaxial trocar with a Hakko 22G needle was inserted, directed to the upper process surface (illustrated in Figures 1D,E), with the needle tip positioned within the foramen (Figure 1F).

The core of the trocar was removed to inject 10 mL of ozone mixed with 10 mL of saline and 1 mL of iodoxanol. This allowed for visualization of the contrast distribution within the spinal canal and around the spinal nerve roots (Figure 1F). A subsequent CT scan was performed post-procedure to confirm the optimal spread of the ozone and contrast agent within the cervical spinal canal, and to check for any presence of bleeding (Figure 1G).

#### 3.2.3 Postoperative care

Immediately following the procedure, the patient was transferred back to the ward and administered intravenous rehydration with a 500 mL sodium chloride solution at a rate of 40 drops per minute. Oral non-steroidal anti-inflammatory medication, Erecoxib (0.1 g), was prescribed twice daily for 3 days post-surgery.

## 4 Observation indicators

### 4.1 Headache symptoms

The frequency of headache episodes (weekly occurrences), the average duration of each episode, the pain intensity (using the Visual Analog Scale [VAS], ranging from 0 to 10) (17), and any accompanying symptoms (such as conjunctival congestion, tearing, nasal congestion, runny nose, etc.) should be tracked and documented before and after the surgery.

### 4.2 Quality of life

The patients’ quality of life should be assessed before and after surgery using the Headache Impact Test (HIT) and additional questionnaires (18). The HIT includes various questions that measure the impact of headaches on daily activities, work, study, and emotional well-being. Higher scores indicate a greater negative impact on quality of life.

### 4.3 Surgical complications

Any complications occurring during and after the surgery, such as bleeding, infection, nerve damage (e.g., upper limb numbness or weakness), cerebrospinal fluid leakage, and other potential issues should be monitored and recorded.

## 5 Follow-up

Patients will undergo regular follow-up evaluations after surgery at 1 week, 1 month, 3 months, and 6 months. These follow-ups will be conducted through outpatient visits, phone calls, and questionnaire surveys. Detailed records will be maintained regarding the patients’ headache symptoms, quality of life, and any surgical complications. Patients are encouraged to promptly report any postoperative discomfort or unusual conditions to facilitate timely medical intervention.

## 6 Statistical analysis

The data was analyzed using statistical software (IBM SPSS 26.0 VERSION). Quantitative data are presented as mean ± standard deviation (x ± s), while categorical data are expressed as percentages (%). The Shapiro–Wilk test was utilized to assess the normality of the distribution. The impact of surgery on headache-related metrics and HIT scores at various time points—pre-surgery, 1 week, 1 month, 3 months, and 6 months post-surgery—was analyzed using repeated measures ANOVA. For multiple comparisons, the Bonferroni correction method was applied to accurately assess changes over these time intervals. A *p*-value of less than 0.05 was considered statistically significant.

## 7 Results

### 7.1 Patient demographics

The study enrolled 17 patients diagnosed with chronic cluster headache who underwent upper cervical spinal nerve root release surgery. The cohort consisted of 12 men and 5 women, ranging in age from 25 to 58 years, with a mean age of 41.5 ± 8.2 years. The duration of their condition spanned 2 to 28 years, averaging 11.7 ± 4.4 years. Prior to the surgery, the frequency of headache episodes varied from 1 to 9 times per week, with an average of 5.4 ± 2.1 episodes. The duration of these episodes ranged from 10 to 120 min, with an average duration of 35.3 ± 21.5 min. The intensity of the headaches, as measured by the Visual Analog Scale (VAS), ranged from 4 to 9, with an average score of 7.29 ± 1.48 (Table 1).

**Table 1:** Demographics characteristics of patients (*n* = 17).

Baseline characteristics	
Mean age (years ± SD)	41.5 ± 8.2 (25–58)
Gender (*n*, %)	
Man	12 (70.6%)
Woman	5 (29.4%)
Duration (years ± SD)	11.7 ± 4.4 (2–28)
Smoking (*n*, %)	
Yes	7 (41.2%)
No	10 (58.8%)
Drinking	
Yes	12 (70.6%)
No	5 (29.4%)
Pain site (*n*, %)	
Left	11 (64.7%)
Right	6 (35.3%)
Combined symptoms, *n* (%)	
Shed tears	14 (82.3%)
Runny nose	15 (88.2%)
Nasal congestion	13 (76.5%)
Conjunctival congestion	16 (94.1%)
Ptosis of eyelids	4 (23.5%)
Facial sweating	2 (11.7%)
Nausea/vomiting	9 (52.9%)
Fear of noise	6 (35.3%)
Afraid of light	4 (23.5%)

Data represent baseline demographic and clinical characteristics of the study cohort (*n* = 17). Continuous variables (age, duration) are presented as mean ± standard deviation (SD), with the range shown in parentheses. Categorical variables are presented as counts (*n*) and percentages (%).

### 7.2 Postoperative pain and quality of life

Six months post-surgery, significant improvements were observed in the management of chronic cluster headache among patients. The average Visual Analog Scale (VAS) score markedly reduced from 7.29 to 1.75. Similarly, the frequency of headache episodes decreased from an average of 5.4 times per week to just 0.76 times. Additionally, the average duration of each episode was reduced from 35.32 min to 4.55 min.

**Figure 2:**
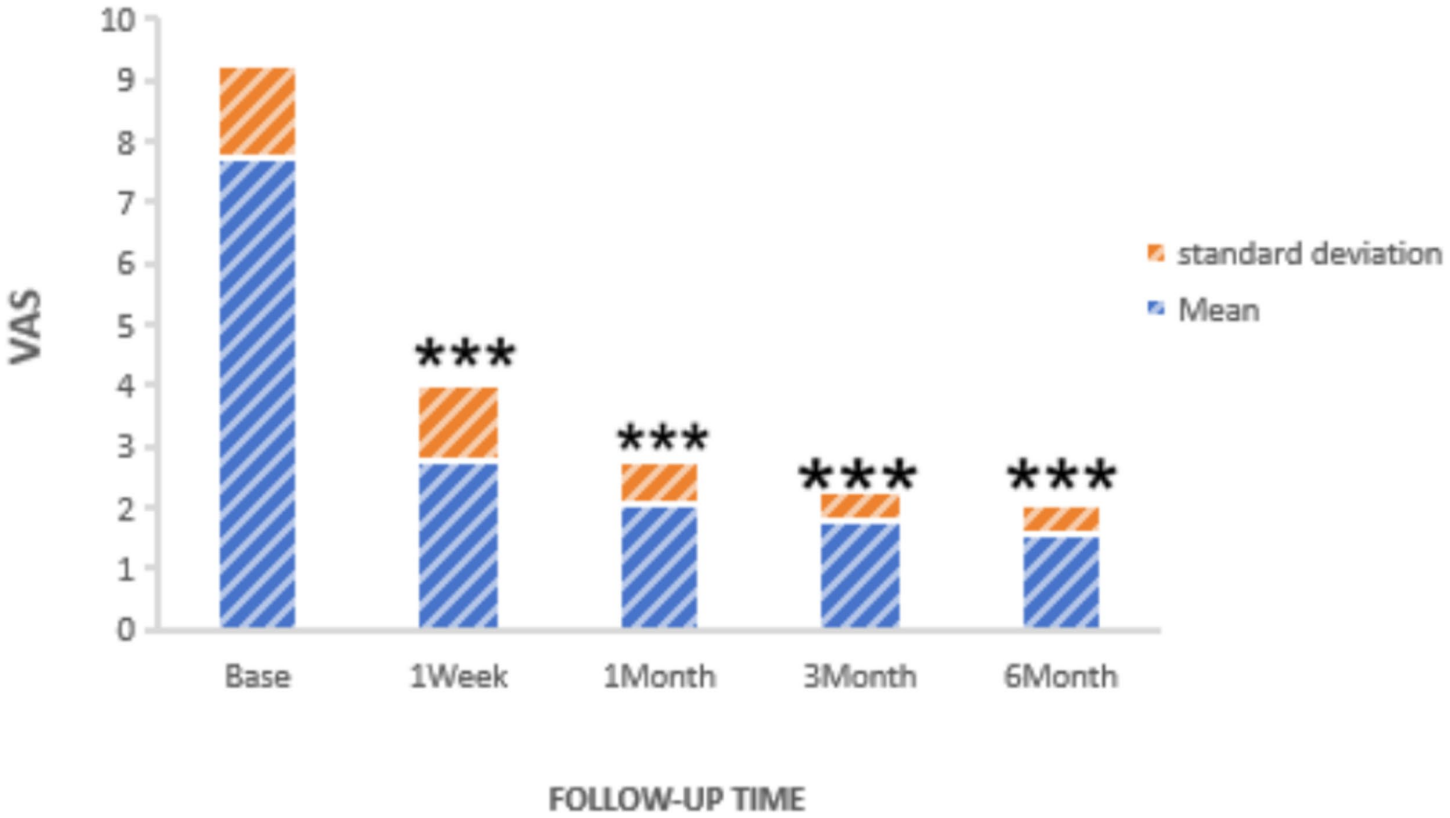
VAS of patients at pre-procedure and each post-procedure time of the follow-up period. ***p < 0.05.

**Figure 3:**
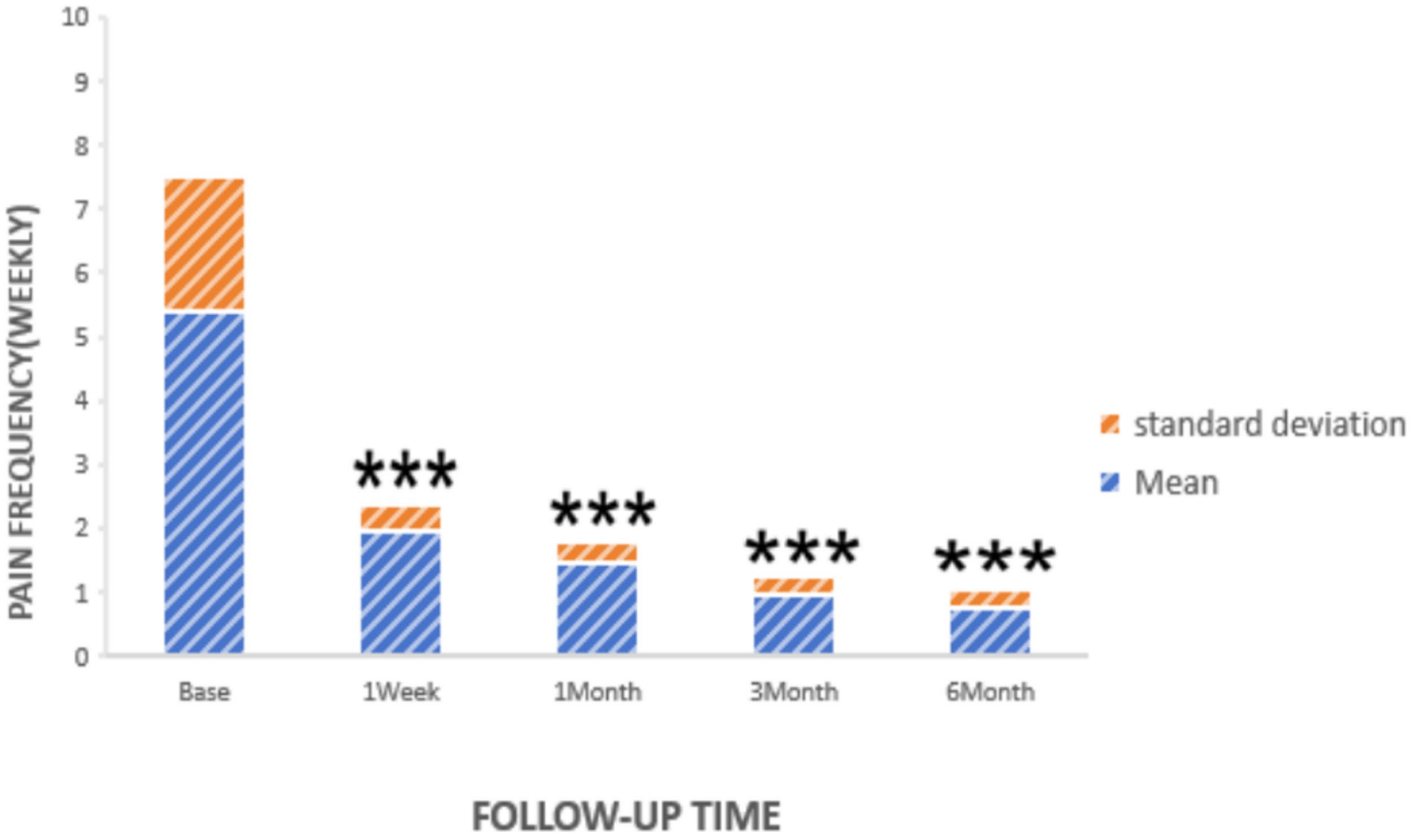
Pain frequency of patients at pre-procedure and each post-procedure time during the follow-up period. ***p < 0.05.

**Figure 4:**
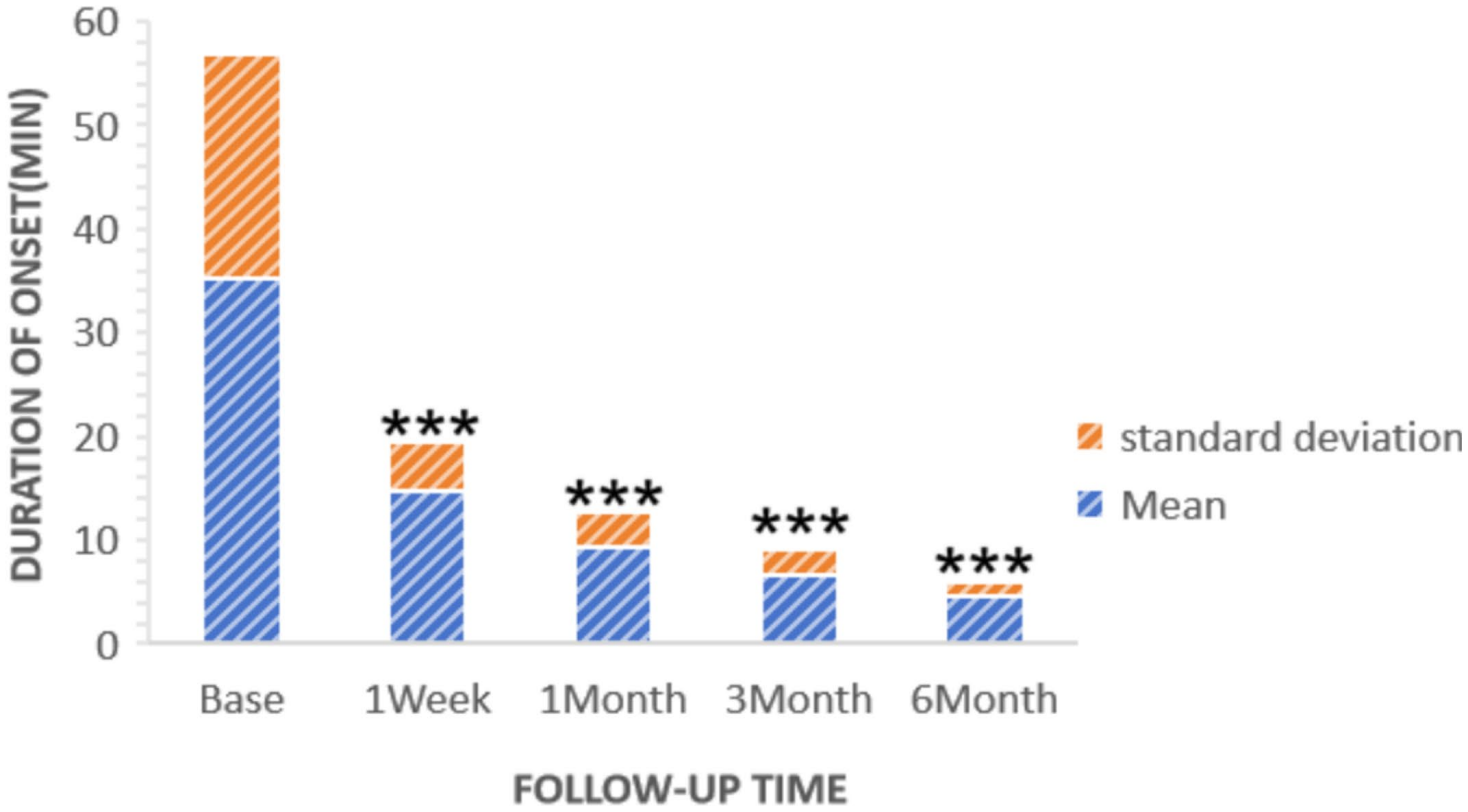
Duration of patients’ onset at pre-procedure and each post-procedure time during the follow-up period. ***p < 0.05.

Statistically significant reductions in VAS scores were noted at all follow-up time points (*p* < 0.05): 1 week post-surgery (2.76 ± 1.16), 1 month (2.25 ± 0.45), 3 months (1.95 ± 0.47), and 6 months (1.75 ± 0.48), as shown in Figure 2. Similarly, the frequency of headache attacks progressively decreased over the 6-month period: 1 week post-surgery (1.96 ± 0.42 times), 1 month (1.45 ± 0.36 times), 3 months (0.95 ± 0.32 times), and 6 months (0.76 ± 0.28 times), detailed in Figure 3. The duration of each headache episode also showed a consistent decrease: 1 week post-surgery (14.68 ± 4.75 min), 1 month (9.44 ± 3.28 min), 3 months (6.65 ± 2.52 min), and 6 months (4.55 ± 1.34 min), as illustrated in Figure 4.

Within 24 h post-surgery, 15 patients experienced significant relief from headaches, while 2 reported unsatisfactory outcomes. Following this, contralateral C3/4 segment surgery was performed 2 days later. Post-operatively, one patient suffered transient vomiting and another experienced numbness in the upper limb; The vomiting patients were relieved quickly after symptomatic treatment with intravenous injection of ondansetron; The numbness in the upper limbs was relieved 1 week after symptomatic treatment.

**Figure 5:**
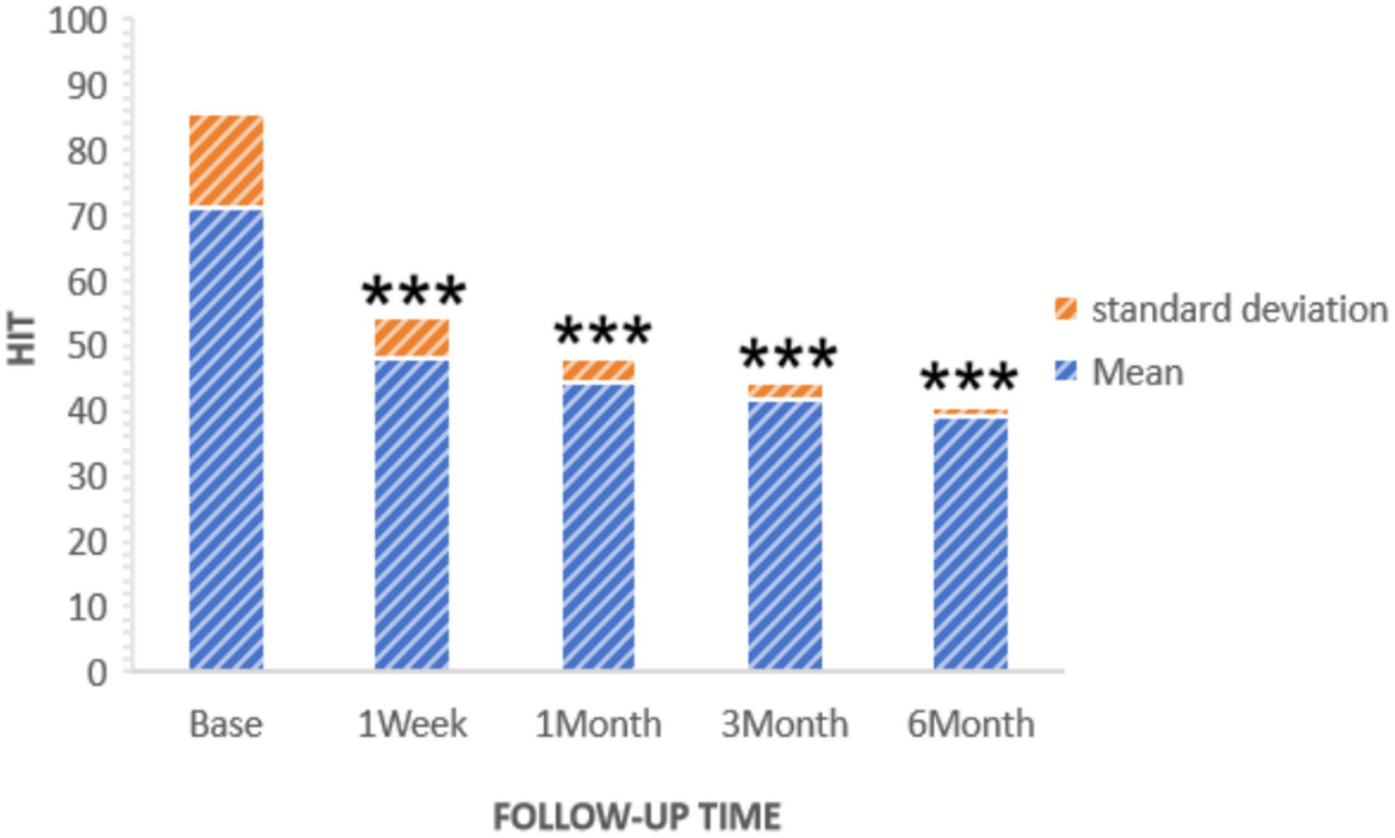
HIT of patients at pre-procedure and each post-procedure time during the follow-up period. ***p < 0.05.

The Headache Impact Test (HIT) was used to assess changes in quality of life. Initially, the average preoperative HIT score was 71.05. Subsequent measurements showed a decrease to 48.06 ± 6.37 1 week after surgery, 44.50 ± 3.46 after 1 month, 41.90 ± 2.62 after 3 months, and 39.43 ± 1.34 after 6 months. These results indicate a substantial and progressive improvement in quality of life, as depicted in Figure 5.

## 8 Discussion

In 2018, the International Headache Society published the “International Classification of Headache Disorders, 3rd Edition (ICHD-3),” which categorizes cluster headaches into episodic, chronic, and probable types (2). These fall under the classification of trigeminal autonomic cephalalgias. Epidemiological studies conducted in the United States and Europe indicate that cluster headaches affect approximately 0.1% of the general population (19). However, prevalence rates can vary across different regions globally. The condition is more common in men, with a men-to-women ratio of around 3:1 (20). The recurrent intense headache episodes experienced during cluster periods significantly impair the quality of life and occupational functioning of affected individuals. In the present study, we explored the efficacy of upper cervical spinal nerve root release surgery as a treatment for cluster headache and obtained positive outcomes. This paper discusses the findings of our research, highlighting the implications and potential for improved patient care in this challenging condition.

### 8.1 Analysis of the results of this study

This study enrolled 17 patients with cluster headache who underwent upper cervical spinal nerve root release surgery. Over a 6-month follow-up period, significant relief in headache symptoms was observed, with notable reductions in frequency, duration, and intensity of pain. Additionally, there was a marked improvement in the patients’ quality of life. By the 6-month follow-up, the symptoms for most patients were essentially under stable control, indicating that upper cervical spinal nerve root release surgery is an effective treatment for cluster headache. Although the sample size is small, the use of repeated measures ANOVA enhances statistical power by analyzing within-subject changes over time. The large effect size observed, coupled with a highly significant *p*-value, suggests that the improvements are clinically meaningful and not merely a statistical artifact.

However, two patients initially did not experience significant pain relief and subsequently underwent contralateral spinal nerve root release, which resulted in considerable improvement. This seemingly paradoxical outcome may be explained by the profound neurophysiological changes that occur in severe, chronic pain states, particularly the key mechanism of central sensitization. In this study, patients with long-standing, refractory cluster headache, the persistent and intense unilateral nociceptive barrage can lead to a state of hyperexcitability within the central nervous system, most notably the trigeminocervical complex (TCC), which serves as the integration center for head and neck pain (21–23). Neuroanatomical and electrophysiological studies provide a solid foundation for this phenomenon. Firstly, the TCC is not a functionally segregated structure but rather a functional continuum where its neurons receive convergent inputs from both the trigeminal and upper cervical spinal nerves (21). Crucially, these neurons receive inputs not only from the ipsilateral side but also from the contralateral side. The existence of transmedian fibers that cross the midline provides a direct anatomical pathway for this bilateral “crosstalk” (24). Under normal physiological conditions, afferent input from the contralateral side is typically sub-threshold and does not elicit a pain response. In a state of central sensitization, however, this changes fundamentally. Evidence from animal models shows that a unilateral inflammatory or nerve injury significantly increases the number of spinal neurons with bilateral receptive fields and can even switch their response from inhibitory to excitatory (25). Therefore, we hypothesize that for these two exceptionally refractory patients, their condition had evolved from a localized unilateral issue to a centralized disorder. The initial ipsilateral surgery addressed the primary driver of pain, but the nervous system remained too sensitized to return to a non-painful state. In this hyperexcitable condition, even minor, previously subclinical irritation on the contralateral side—such as the subtle nerve root irritation we noted upon imaging review—was sufficient to perpetuate the hyperexcitability of the TCC and maintain the pain cycle. The subsequent contralateral procedure was likely effective because it eliminated this secondary, yet critical, nociceptive input, finally breaking the pathological cycle and allowing the sensitized central nervous system to quiet down.

### 8.2 Mechanism of surgical treatment for CCH

Upper cervical spinal nerve root release surgery may offer relief through several mechanisms. First, there is neurological function regulation. The surgery potentially influences nerve signal transmission in headache-related structures, such as the trigeminal spinal tract nucleus and the hypothalamus, thus modulating neurological functions and diminishing headache symptoms (26, 27). Studies indicate that cluster headaches involve abnormal activation of the trigeminal neurovascular system, which results in vasodilation and neurogenic inflammation. The trigeminal cervical complex, a pivotal anatomical and functional entity in the pathogenesis and management of refractory headaches, simultaneously receives afferent fibers from the trigeminal nerve and superior cervical spinal nerve (C1-C2) and promotes the transmission and regulation of pain signals (8–11). Surgical decompression of the nerve roots in the upper cervical spine may thus reduce trigeminal nerve excitability and alleviate pain. Second, you can enhance local blood circulation. The surgical intervention may enhance blood flow around the cervical nerves, decrease the release of inflammatory mediators, and thus mitigate headache symptoms. Research by Bakhtadze et al. proposes that spinal joint dysfunction can lead to cerebral hypoperfusion via overactivity of the regional sympathetic nervous system, potentially elucidating the pathogenesis of certain intractable headaches (28). In this study, the surgical release of spinal nerve roots can effectively relax local sympathetic nervous tension, boost blood circulation, and consequently aid in reducing neuroinflammation and pain.

The potential pharmacological mechanisms of transforaminal upper cervical epidural ozone injection in this surgical procedure include the following. First, there is the neuromodulatory effect, where the oxygen-ozone mixture, characterized by strong oxidative properties and excellent tissue diffusibility, induces mechanical separation of perineural adhesions (29). Second, there is nociceptive modulation, where ozone selectively inhibits the release of nociceptive neurotransmitters, particularly reducing concentrations of substance P and calcitonin gene-related peptide (CGRP), thereby attenuating peripheral sensitization processes (30). Third, there is the anti-inflammatory cascade, where ozone exerts dual anti-inflammatory actions through suppressing synthesis of proteolytic enzymes and pro-inflammatory cytokines (e.g., TNF-*α*, IL-6), while activating the Nrf2 pathway to enhance antioxidant enzyme expression. This combined effect effectively neutralizes oxygen free radicals and preserves neural structural integrity, ameliorating neurogenic inflammation during cluster headache episodes (31, 32). Fourth is neuronal regulation, where ozone activates inhibitory interneurons in the spinal dorsal horn, promotes the release of endogenous analgesics (e.g., enkephalins), and modulates neuronal excitability through sodium-potassium channel regulation, collectively modifying pain transmission pathways (33).

In terms of safety, only minor complications were reported. One patient experienced transient vomiting, and another reported temporary upper limb numbness post-surgery; both conditions resolved within a week with symptomatic treatment. No other significant complications were noted, underscoring the high safety profile of the surgery.

### 8.3 Future research directions

(1) Expand sample size

Conduct multi-center, large sample studies to more accurately evaluate the efficacy and safety of surgery.

(2) Long-term follow-up

Further observe the long-term effects and recurrence of surgery, as well as the long-term impact on the patient’s quality of life.

(3) Comparative study

We recognize the importance of further verifying the efficacy of upper cervical nerve root release surgery. One potential approach may be to conduct multiple-center, randomized controlled trials with a placebo group to better understand the mechanism of action of the surgery and further support its efficacy.

## 9 Conclusion

This study underscores the efficacy and safety of upper cervical spinal nerve root release surgery in managing cluster headaches. The findings reveal that the surgery substantially reduces headache symptoms and enhances patients’ quality of life. Importantly, no significant postoperative complications were reported, affirming the procedure’s safety. The initial success of this surgical technique introduces a promising new treatment alternative for cluster headache sufferers. It stands out as a viable option that offers patients a low-risk and efficient pathway to recovery, making it a compelling choice for those grappling with this debilitating condition.

## Data Availability

The original contributions presented in the study are included in the article/supplementary material, further inquiries can be directed to the corresponding authors.
